# Insect Detection of Small Targets Moving in Visual Clutter

**DOI:** 10.1371/journal.pbio.0040054

**Published:** 2006-02-07

**Authors:** Karin Nordström, Paul D Barnett, David C O'Carroll

**Affiliations:** **1**Discipline of Physiology, School of Molecular and Biomedical Science, The University of Adelaide, Adelaide, Australia; University of SussexUnited Kingdom

## Abstract

Detection of targets that move within visual clutter is a common task for animals searching for prey or conspecifics, a task made even more difficult when a moving pursuer needs to analyze targets against the motion of background texture (clutter). Despite the limited optical acuity of the compound eye of insects, this challenging task seems to have been solved by their tiny visual system. Here we describe neurons found in the male hoverfly,*Eristalis tenax,* that respond selectively to small moving targets. Although many of these target neurons are inhibited by the motion of a background pattern, others respond to target motion within the receptive field under a surprisingly large range of background motion stimuli. Some neurons respond whether or not there is a speed differential between target and background. Analysis of responses to very small targets (smaller than the size of the visual field of single photoreceptors) or those targets with reduced contrast shows that these neurons have extraordinarily high contrast sensitivity. Our data suggest that rejection of background motion may result from extreme selectivity for small targets contrasting against local patches of the background, combined with this high sensitivity, such that background patterns rarely contain features that satisfactorily drive the neuron.

## Abstract 2

Visual neurons in male hoverflies respond selectively to small moving targets, even in the presence of cluttered backgrounds, suggesting exquisite feature detection properties.

## Introduction

Visual detection of targets that move within visual clutter is a common task in animals searching for prey or conspecifics. In birds of prey such as kestrels, for example, astonishing optical acuity permits visual detection of prey at distances of up to 275 m from a perch [1]. This task becomes even more challenging if the pursuer is itself in motion so that targets need to be analyzed against the motion of background texture (i.e., against moving clutter). Despite the limited optical acuity of a compound eye, the challenging task of target detection in visual clutter seems to have been solved by the tiny visual system of insects, making them excellent models for studying higher order visual properties [2]. Among the insects, hoverflies stand out with their highly sophisticated behavior of acrobatic aerial maneuvers and interactions among conspecifics that necessitate visual detection of targets under a broad range of conditions [3–6].

Neurons optimized for the detection of objects that move relative to the surround have been described from the lobula complex of several insect groups [7–9]. In dipteran flies, target-selective neurons have been described in blowflies [9,10] and hoverflies [4]. However, little is yet known about their physiology or the mechanisms underlying their tuning. We describe here a class of neurons in the hoverfly,*Eristalis tenax,* which are tuned to the detection of small moving targets. We show that these neurons continue to respond selectively to the motion of small targets under a variety of confounding background motion stimuli. This is a property that has not previously been described for any insect neuron.

## Results

### Neuron Classification

We recorded from neurons in the lobula complex of 74 male hoverflies that were strongly excited by the motion of small, black targets (0.8° square) within a fronto-dorsal receptive field (Figure 1). As in some insect neurons described previously [3,8], large targets (>3°) elicit weaker responses, which fell to spontaneous levels for targets subtending more than 10° (Figure 2). We recorded from 206 neurons meeting these criteria, which we therefore classify as small-target motion detectors (STMDs).

**Figure 1:**
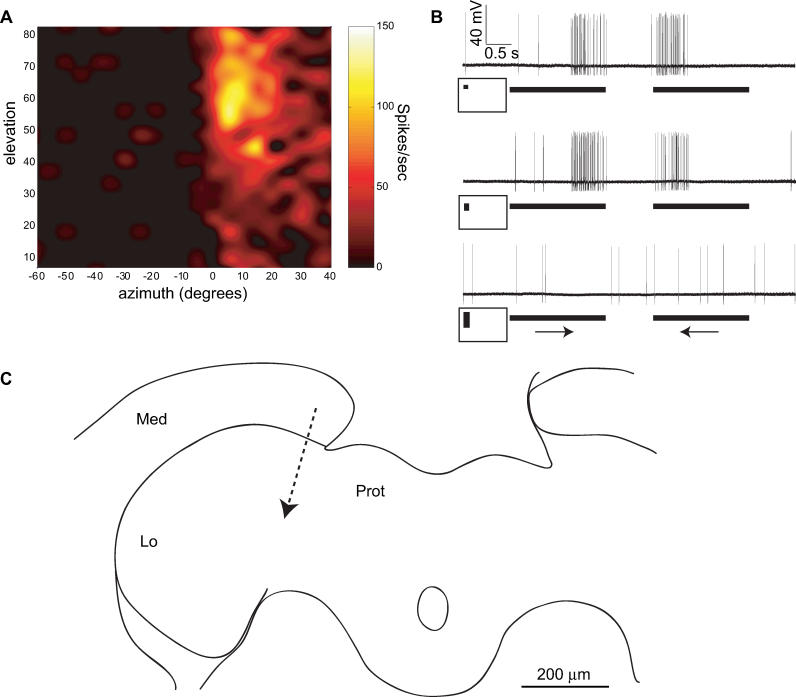
Physiology and Morphology of a Typical Class of Non-Directional STMD (Type 1) with a Large Contralateral Receptive Field, Recorded from the Left Lobula (A) The receptive field map constructed by drifting targets in four directions across the stimulus display (seeMaterials and Methods). Elevation values are positive above the equator, and azimuths are negative to the left of the midline (i.e., ipsilaterally). (B) Raw responses of the neuron to motion of three different-sized targets (0.8°, 3°, or 15° high by 0.8° wide) drifted through the center of the receptive field (horizontal scans at an elevation of 60°). The bars indicate the peri-stimulus duration and the arrows the direction of target motion. (C) The morphology of STMD 1 reconstructed from a Lucifer yellow fill shows extensive arborization through the lobula (Lo). A contralateral projection through the protocerebrum (Prot) probably provides the input. The arrow points to the recording location. Med, medulla.

**Figure 2:**
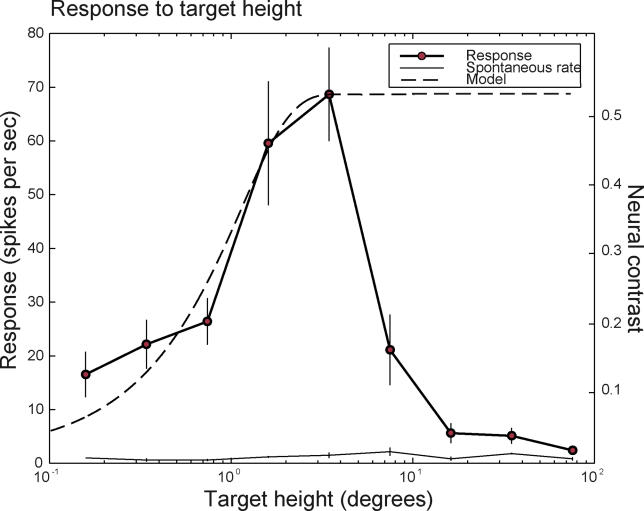
Length Tuning of STMD 1 Neurons Sharply Tuned to Small Features The strongest spiking response is seen to small bars (0.8° × 3° of the visual field). Larger bars moving through the receptive field elicit no response. Error bars show standard error of the mean of experiments in different neurons of the same STMD class (*n* = 9). The dashed line shows the output of a model for apparent contrast of targets of different size, as viewed by a single photoreceptor centered on the target (seeMaterials and Methods). Close fit between the model output and the lower part of the neuron response tuning suggests that attenuation of responses to targets below the optimum is due to reduction in their apparent contrast, not the smaller size per se.

The STMD neurons include numerous classes: We further (and somewhat arbitrarily) divided the recorded neurons into 20 classes on the basis of differences in receptive field location, size, and direction selectivity. We have stained a small number of neurons from this group by iontophoretic injection of fluorescent markers, and although some of the anatomically identified neurons resemble male-specific target-sensitive neurons described from other fly species [9], our staining also identifies several unique neurons that have not previously been described in flies (e.g.,Figure 1). Until we have a complete functional and anatomical description of the entire element of what are clearly a very diverse group of neurons, it is premature to give the neurons functional names in this paper. Instead, we focus here on categorizing STMD neurons on the basis of responses to the motion of targets against that of large optical flow stimuli. We have however labeled two of the neuron classes (STMD 1 and 2), described in more detail in the text.

Figure 1 shows responses of a typical STMD (STMD 1). Stable recordings from healthy STMDs typically (but not always) lack spontaneous firing activity, but when a small target moves through their receptive field, they give a powerful response that is often in excess of 200 spikes/s for optimal-sized stimuli. In many cases (as inFigure 1B), the response of STMDs to larger stimuli is indistinguishable from spontaneous activity.

### Responses in Visual Clutter

Existing models for insect feature detection suggest that selectivity for small targets arises from inhibitory feedback from “tangential” neurons [10–13] sensitive to wide-field optical flow and found in a specialized sub-region of the lobula complex, the lobula plate. For example, the FD1 neuron of the blowfly lobula plate has been shown to receive inhibitory feedback via GABAergic synapses with the outputs of centrifugal horizontal (CH) tangential neurons tuned to the motion of wide-field optical flow stimuli [11,13]. A prediction of such models is that the presence of moving background “clutter” should inhibit the response to target motion. To test whether the STMD neurons we describe are tuned by similar mechanisms, we designed a random, broadband pattern as a background “clutter” stimulus (Figure 3A) that strongly excites lobula plate tangential neurons. We confirmed the effectiveness of the background pattern as a stimulus for wide-field motion sensors by recording the response from graded horizontal system (HS) neurons in the lobula plate.Figure 3B shows the response of HSNE neurons to motion in the preferred direction. The neurons respond to the motion of the pattern with a large graded depolarization, similar to those obtained with an optimal sinusoidal grating pattern in earlier studies (e.g., [14]). We recorded from eight of the 20 STMD classes on over 80 occasions, 30 of which gave us useful data in the presence of this background-motion stimulus. The majority of neurons (six out of eight classes) gave no response (Figure 3C), either inhibitory or excitatory, to the background texture. While the lack of excitation is hardly surprising for neurons tuned to small targets, the absence of inhibition suggests that they do not receive inhibitory feedback from tangential neurons.

**Figure 3:**
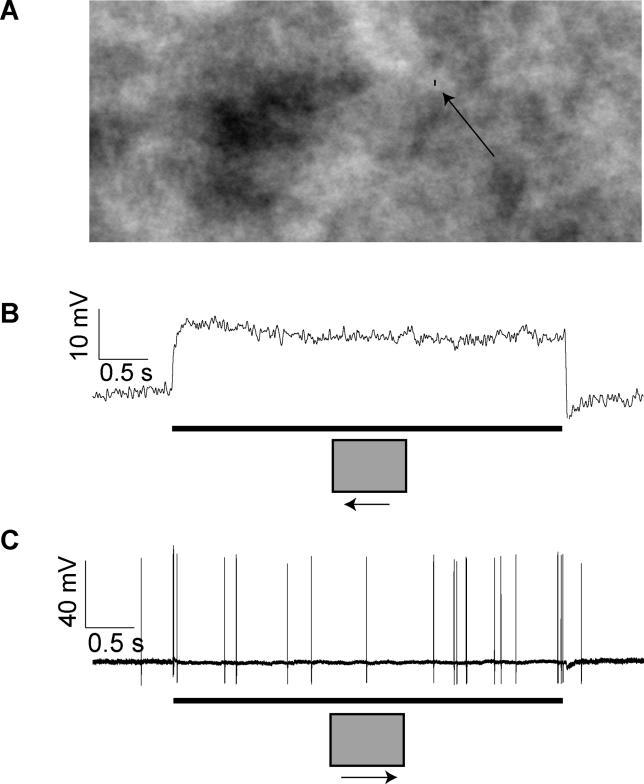
The Background Clutter Pattern Used in Figure–Ground Experiments The pattern used in figure–ground experiments (A) contains components of random frequency and phase, with 1/f statistics similar to those of natural scenes. It excites HS tangential neurons maximally (average of five recordings [B]), but has no effect on most STMDs (STMD 1 [C]). The bars indicate peri-stimulus duration and the arrows the direction of clutter motion.

When we drift a small target across the receptive field of five of these six neuron classes, including both direction-selective and non-directional units, the response to target motion was remarkably robust in the simultaneous presence of this background motion (15 out of over 60 recordings). We obtained reliable recordings from STMD 2 on at least ten occasions, and therefore selected this class for detailed analysis.Figure 4 shows data for a single neuron of this class. The neuron is strongly direction selective (Figure 4A), preferring motion of targets downward and to the left (the neuron being recorded in the left lobula complex). Dye-filling with Lucifer yellow reveals a morphology remarkably similar to that of HS tangential neurons (Figure 4B), albeit with a different preferred direction of motion. Like HS tangential neurons, the neuron has a relatively large axon diameter and a cell body located in lateral mid-brain. Confocal images show the dendritic arborization in the lobula complex to be confined to the dorsal lobula plate. Its output region in the subesophageal ganglion overlaps that of HS tangential neurons [15] and could thus provide input to descending neurons connecting to flight muscles. Overall, the morphology and receptive field location of this neuron are similar to that of certain of the so-called male-lobula-giant (MLG) neurons in blowflies, and of the MLG2 neuron in particular [9]. The compact input arborization and physiological receptive field (Figure 4A and4B) could hardly be described as giant, however. Physiologically, the response of this neuron is distinctive, with vigorous spiking responses to target motion superimposed on a strong graded depolarization (Figure 4C).

**Figure 4:**
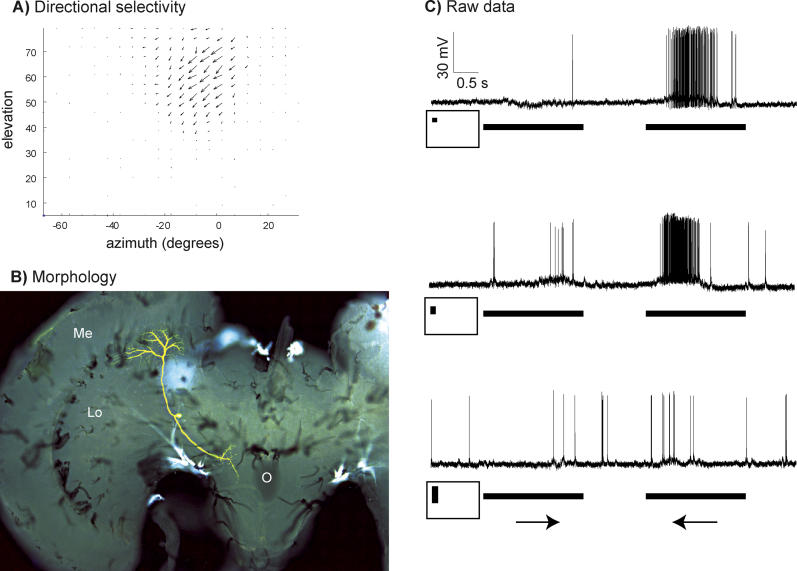
Physiology and Morphology of a Direction-Selective STMD Neuron Class Selected for Detailed Analysis (STMD 2) (A) A receptive field map of local preferred direction. The neuron responds optimally to motion down and away from the midline. The length of the arrows indicates the strength of the response, and its angle the preferred direction of motion (seeMaterials and Methods). (B) A*z-*axis projection of the morphology of STMD 2 after Lucifer yellow staining. A compact group of dendrites in the dorsal lobula plate correspond closely to the retinotopic location of the physiological receptive field and are presumably inputs. Outputs are located in the lateral subesophageal ganglion. Lo, lobula; Me, medulla; O, esophageal foramen. (C) Raw responses to target stimuli drifted against bright backgrounds as inFigure 1.

In several other recordings from other neurons (not identified anatomically) with otherwise similar physiological receptive fields, responses show biphasic action potentials and no graded response (Figure 5), so the STMD 2 “class” could actually include several distinct neuron types.Figure 5 shows responses from STMD 2 to several different figure–ground stimuli. The neuron responds robustly in the presence of background motion in either the preferred or anti-preferred direction for the target motion. InFigure 5B and5C, the background pattern (45°/s) moved slightly slower than the target (50°/s), with the position of the background texture randomized between presentations. Velocity differences have been considered an essential cue for discrimination of moving features [10,12,16,17]. However, the response persists even when we match the velocity of the background pattern to that of the target (50°/s,Figure 5D).

**Figure 5:**
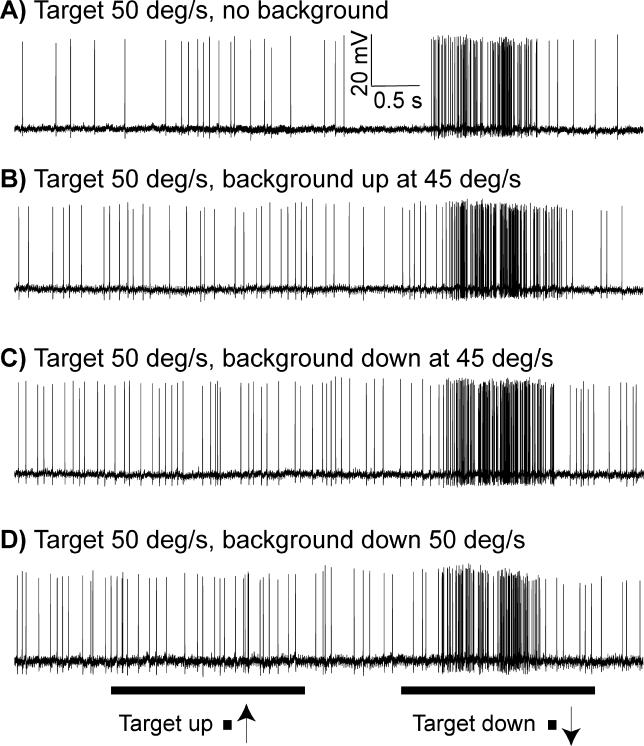
Responses of STMD 2 to Targets Drifted against Cluttered Backgrounds (A) Control experiment showing the sharp response to downward motion of targets (at 50°/s) as they cross the center of the receptive field. Addition of motion of a background texture moving either upwards (B) or downwards (C) at slightly lower speed (45°/s) has little effect on the response. Even when the background is drifted at the same speed (50°/s) and direction as the target (D), the neuron continues to respond.

### Contrast Sensitivity

Our results are surprising because the feedback hypothesis predicts that the response to targets should be strongly suppressed by the presence of background motion [10–12]. This property is not universal among the STMD neurons we recorded however: The presence of moving background clutter inhibited the response to target motion in one of the eight STMD classes we studied (three recordings). In the five classes referred to earlier however (e.g.,Figure 5), the response to target motion was remarkably robust in the presence of confounding background motion. Could this response result from the high contrast of the target against the background pattern? To the human eye, the target is obvious in even a static view of the stimulus (Figure 3A) because the random phase of individual components of the background pattern we selected leads to an image that lacks hard edge contours, whereas the target has sharp black edges. The low resolution of typical insect optics would [18], however, blur the boundaries of the target considerably, so detection of the target against the cluttered background requires extraordinary contrast sensitivity. The 0.8° by 0.8° target size selected in these experiments is a powerful stimulus for STMDs (Figures 1B and4C), yet is well below the size of the receptive field of just a single photoreceptor in the eye of*E. tenax.* Indeed, an optical model that takes account of the modulation transfer function of the eye and the stimulus size (Figure 2) suggests that such small targets have an effective (“neural”) contrast against even the brightest parts of the background of just 20%.

We have further quantified the limits that effective contrast places on the target in two ways for these neurons.Figure 6 shows data from six STMD 2 neurons. Our optical model (Figure 2) provides a close fit to the lower part of the neuron response tuning to small targets, suggesting that attenuation of responses to targets below the apparent optimum is due to reduction in their apparent contrast by spatial blur, not the smaller target size per se. If we use this optical model (Figure 2) to predict the effective contrast of targets below the size of a single photoreceptor, moved against a bright background, we can thus construct a response/contrast function. Remarkably, even the smallest targets used in this analysis, just a single pixel high (0.2° high by 0.8° wide), with an effective contrast of 12%, still produce responses at 50% of the maximal response and significantly (*p* < 0.05) above spontaneous levels (Figure 6A). In some neurons we have even observed responses significantly above spontaneous levels (*p* < 0.05) to single pixel targets (0.2° square). Given that such targets have an effective neural contrast of just 1%, this shows that their contrast sensitivity rivals the highest published for any visual neurons in insects [19] and indeed rivals the psychometric contrast sensitivity of human observers viewing optimal large-field motion stimuli [20].

**Figure 6:**
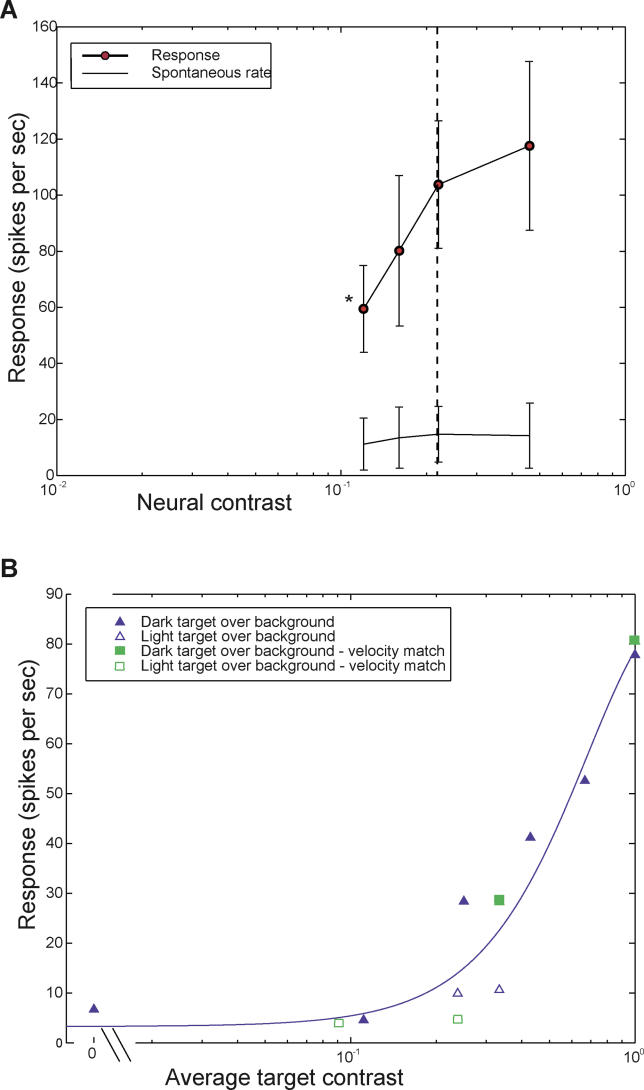
Contrast Sensitivity of STMD 2 (A) The response to targets drifted against a bright background, plotted against the effective (“neural”) contrast of targets smaller than the receptive field of individual photoreceptors (see text). Data show the mean and standard error for six recordings in different animals. The dashed line denotes the effective (i.e., maximum) contrast of the targets used in (B) in which sensitivity to targets of different luminance was evaluated in the presence of confounding background motion moving in the same direction as the target. We varied the luminance of targets moving at 50°/s to be both brighter (open symbols) and darker (filled symbols) than the average of the background texture, under two conditions: moving across the background moving at 45°/s (blue triangles), or with the velocities matched at 50°/s (green squares). The graph shows the mean of three recordings from a single neuron, plotted against nominal average Michelson contrast. The solid line shows a least squares fit of a normalized Weibull function fitted through all conditions.

In the second set of experiments, we directly manipulated the gray level of the target against the background clutter pattern, with either different or matched velocity for target and background while moving in the same direction (Figure 6B). Because the background luminance of this stimulus is variable locally, the contrast of the stimulus can only be expressed in an average sense, which makes comparison with the contrast sensitivity against a plain background difficult. Nevertheless, it is clear that contrast sensitivity remains remarkably high independent of the type of background motion present.

Figure 7 shows a summary across five STMD classes, including STMD 1 and 2 described in more detail inFigures 1 and4. Although we found responses to matched velocity stimuli to be more variable than when a velocity difference was present (Figure 7D), average responses were not significantly different from those observed in the absence of background patterns (e.g.,Figure 5). This suggests that responses might depend more on which features of the background pattern were present within the receptive field than on the background motion per se. Indeed, responses to the target were only significantly reduced when the target luminance was lowered to better match that of the background (Figure 7E and7F).

**Figure 7:**
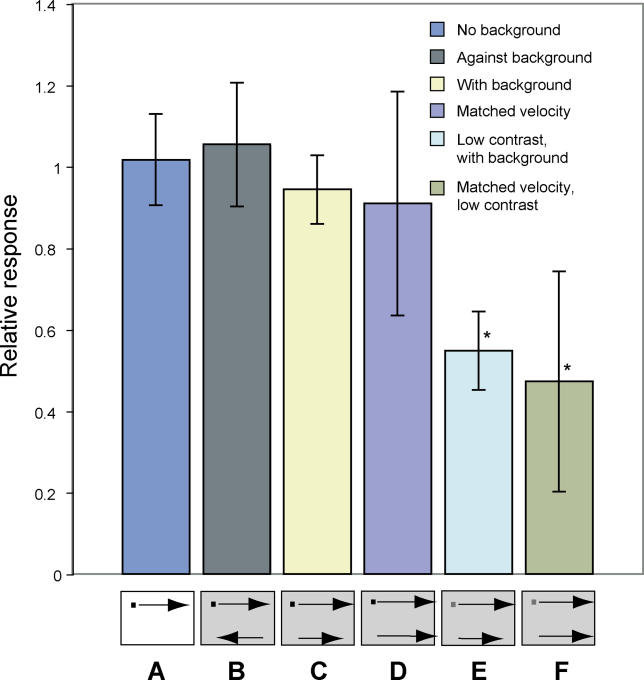
Summary of Data from Five Classes of STMDs (including Type 1 and 2) to Target Motion in the Preferred Direction Responses were estimated from the period during which the target was within the receptive field and normalized to a control (A) in which no background was present (error bars are 95% confidence limits). Responses to targets presented against moving backgrounds were independent of the speed and direction of the background ([B–D] corresponding toFigure 5B–5D), although variance increases if background speed is matched to the target (D). Significant reductions (*) in response were observed when target contrast was reduced either with a speed differential ([E]*t* = 13.2755,*p* < 0.001) or with matched speed ([F]*t* = 6.0156,*p* < 0.05).

### Spatial Inhibition and Response Tuning

In some STMDs, powerful inhibition (in the form of discrete IPSPs [inhibitory postsynaptic potentials] or graded hyperpolarization) may be evident in responses to larger targets (Figure 8). The absence of graded responses in other neurons may reflect the recording site: The neuron shown inFigure 1 had a contralateral receptive field, and although we were unable to locate the soma, the long, narrow axon crossing the mid-line of the brain makes it probable that the response originated contralaterally, so that any graded signals had decayed away by the time action potentials reached the recording site (on the medial margin of the lobula complex, arrow inFigure 1C). Interestingly, where graded or mixed-mode responses are evident, we only observe inhibition to spatially discrete objects such as moving bars. Large-field grating stimuli moving at similar speed to the discrete target stimuli produce no response at all, either excitatory or inhibitory, suggesting that if feedback from wide-field motion-sensitive neurons is involved in shaping responses to target motion, it must be operating pre-synaptically (Figure 8). The clear inhibition by elongated bar stimuli indicates that response tuning is more complex than the general feedback model [10–13], since it suggests the presence of at least a second, post-synaptic spatial inhibitory mechanism, in addition to whatever feedback (or feedforward) process generates pre-synaptic inhibition.

**Figure 8:**
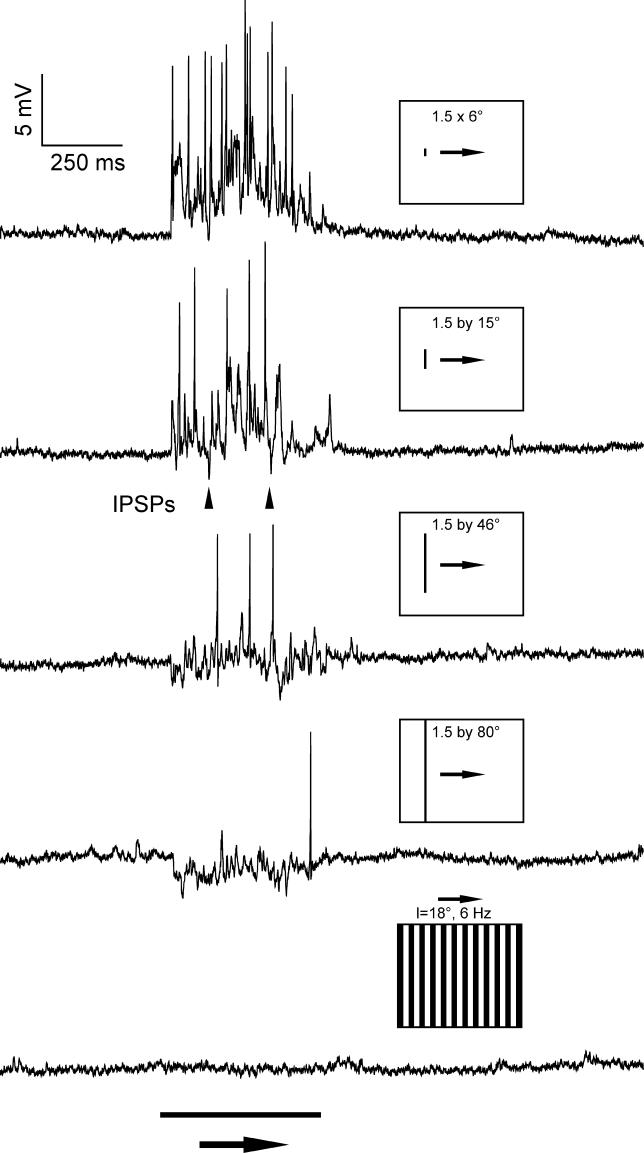
Complex Spatial Inhibition in an STMD Neuron Displaying Mixed Graded/Spiking Responses Small targets elicit excitatory postsynaptic potentials (EPSPs) and monophasic spikes riding on a net graded depolarization. As length of a bar stimulus is increased, discrete IPSPs (arrowheads) become evident, eventually summing to produce net hyperpolarized responses for long bars. In each case, the bar stimuli were drifted through the receptive field at 75°/s. A drifting grating stimulus with similar spatio-temporal properties (temporal frequency 6 Hz, spatial wavelength 18°) elicits no post-synaptic potentials.

### Response to Background Features

Clearly, if these neurons can respond to motion of a target moving at the same velocity as the background, there will be certain patterns of wide-field motion that could contain features that elicit a response. Such instances could be rare in normal visual behavior however, since these neurons described responded only when an optimal-sized target moves in the appropriate direction and within a relatively small receptive field. However, we found two classes of STMDs that are an exception to this rule, giving a strong response to the clutter-only stimulus (seen in five separate trials, out of 15 recordings on these classes), despite sharp response tuning to small targets when tested with elongated bars (Figure 9A). Interestingly, although the response to a small target (Figure 9B) is a sustained, intense burst of activity as the target crosses the receptive field, the response to the wide-field clutter stimulus (Figure 9C) is weaker and variable over time, suggesting that it results from specific features within the random pattern successively entering and exiting the receptive field, rather than the global motion of the background stimulus. Further experiments with more stereotyped background patterns would be required to determine which specific features of the pattern the neuron responds to.

**Figure 9:**
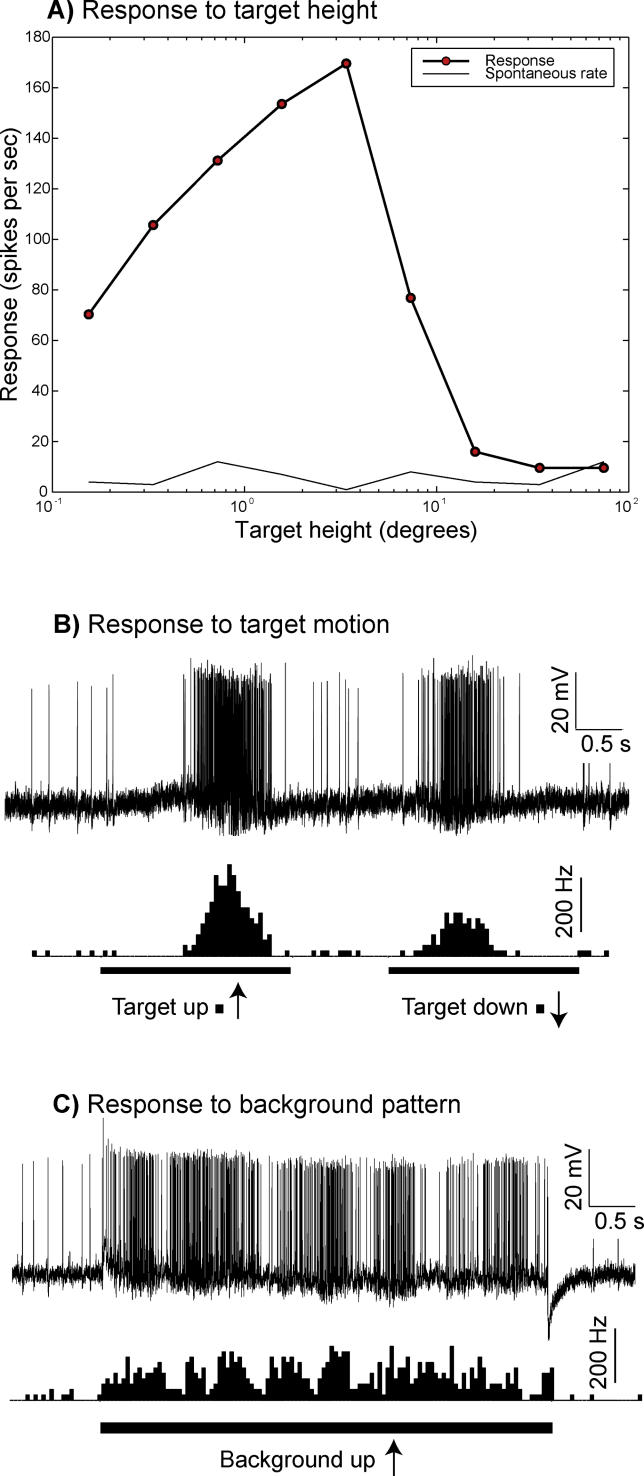
Responses of a Weakly Direction-Selective STMD Neuron That Responds to the Visual Clutter Stimulus (A) Length tuning to discrete bar stimuli (seeFigure 2) is typical of STMD neurons, with clear tuning to small features. (B) Raw responses to a target drifted in alternating directions show a vigorous, sustained response as the target crosses the receptive field. (C) The clutter stimulus (Figure 3A) produces an erratic and weaker response.

## Discussion

The robust rejection of background motion by many STMD neurons may result, in part, from the extreme selectivity of these neurons for small, contrasting features (Figure 2) and the very distinctive receptive-field properties (both location and direction selectivity). The spatial tuning of these neurons is remarkable given the relatively low-resolution optics of insect compound eyes [18]. The sharp roll-off in the neural response to target sizes above 3° suggests that the neural pathway processing small-target motion incorporates powerful and local spatial inhibition on the scale of the photoreceptor mosaic. This could, in turn, provide at least a partial basis for rejecting background clutter, since background scenes may rarely invoke the appropriate combination of cues.

This is clearly not the only process at work however: Large grating patterns fail to invoke either excitation or post-synaptic inhibition, even though such inhibition is often evident when large discrete targets move across the receptive field (Figure 8). This suggests that at least two distinct forms of inhibition shape the response tuning to small targets, operating at different stages in visual processing. The pre-synaptic mechanism might operate via feedback from tangential optic flow neurons to early stages of processing. Further experiments with a broad range of stimuli that test the degree to which responses remain robust in the presence of nearby “distractor” stimuli moving in a similar direction to the target are required to further elucidate the mechanism for this complex tuning.

STMDs such as those described here could be an appropriate adaptation for the tracking of females that might be moving either against bright skies or cluttered foliage [3–5]. Hoverflies are easily recognized by their ability to hover mid-air, apparently stationary. Following detection of mates or rivals, they switch to spectacular high-speed pursuits, mediated by visual cues [4,21]. Neural circuitry that permits higher-order target neurons to respond to targets while remaining oblivious to background motion would be advantageous for object discrimination under different surround conditions. Identification of such neurons in the lobula complex of flies provides an exciting basis for developing models for such complex behavior.

## Materials and Methods

### Experimental setup

Wild-caught male hoverflies*(Eristalis tenax)* were immobilized with wax and mounted in front of an RGB CRT visual display with a high refresh rate of 200 Hz and a mean luminance of 150 cd/m^2^. Visual stimuli were presented using Vision Egg software (http://www.visionegg.org). Neurons were recorded intracellularly using aluminum silicate micropipettes pulled on a Sutter Instruments P-97 puller and filled with 2 M KCl. Electrodes had a typical tip resistance of 120 MΩ. The display subtended 100° × 75° of the fly's visual field of view, with a resolution of 640 × 480 pixels, permitting targets down to about 0.2° square to be presented. Data were digitized at 5 kHz using a 12-bit A/D converter (National Instruments, Austin, Texas, United States) and analyzed off-line with Matlab (http://www.mathworks.com).

### Neuron characterization

Upon penetration of the neurons, we subjected each to a succession of stimuli in order to categorize its response to different classes of motion. Receptive fields (Figure 1A) and direction selectivity (Figure 4A) of neurons that responded to small targets were determined using a series of 21 vertical and horizontal scans with a black 0.8° square target moving at 50°/s across the bright CRT display. Subsequently, targets were presented moving in a single path across the center of the receptive field. We defined neurons as STMDs when the response was selective for small targets, determined using a series of bars of variable height (Figure 2) and the width fixed at 0.8°. Typical STMDs gave highly selective responses to small moving targets subtending only a few degrees of the visual field, but no response to larger targets or wide-field stimuli, such as sinusoidal gratings. We recorded from 206 STMD neurons in 74 male hoverflies.

### Morphology

To identify recorded neurons, in some cases we backfilled micropipettes with 4% Lucifer yellow in 0.1 M LiCl. The dye was then injected by passing a hyperpolarizing current (0.2 to 2 nA, depending on the amount of current individual electrodes would pass without blockage) for 1–10 min. Following electrophysiology, the brain was dissected out of the head capsule, fixed in 4% paraformaldehyde (in 0.1 M phosphate buffer), dehydrated through an ethanol series, and cleared in methyl salicylate. A series of digital photographs were taken at different depths of the tissue, and the morphology of the neuron was reconstructed using Adobe Photoshop (San Jose, California, United States). Position of the neuron within the lobula complex (Figure 4B) was confirmed by constructing a three-dimensional image from a Z-series in a scanning confocal microscope (MRC-1000UV; Bio-Rad, Hercules, California, United States).

### Responses to figure–ground stimuli

By exchanging the bright screen with a textured background, we could analyze the response to targets in clutter. We developed a random texture pattern by summing Gabor wavelets with random orientation and phase and with a spatial frequency and contrast distribution that yields an image with a similar two-dimensional power spectrum to that of typical natural scenes and a mean luminance equal to the mid-point in the grayscale lookup table (Figure 3A). Targets were displayed moving at 50°/s. The background was either moving at 45°/s or matched to that of the target.

### Target contrast

Most experiments were conducted using negative contrast, i.e., black targets on a bright background (luminance approximately 300 cd/m^2^) with a nominal contrast of 1.0. In some experiments, we altered target contrast by increasing the luminance of the target closer to that of the mean luminance of the background clutter pattern (i.e., a gray value of 0.5). It is difficult to define contrast of target against visual clutter, because it varies over time as the target moves against different features of the background pattern. However, we quantified the average contrast using the Michelson definition, i.e.:



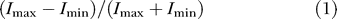



where maximum or minimum values may be the luminance of either target or the mean background luminance in either negative or positive contrast stimuli. Although a black target will always have a contrast of 1.0, a white target (gray value of 1.0) over the background clutter (0.5) thus has an average contrast of 0.33:



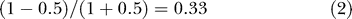



### Analysis

Analysis of spiking responses was carried out off-line in Matlab by band-pass filtering the digitized response and then detecting spikes using an algorithm that makes use of both edge and relative magnitude (level) cues. Receptive fields were obtained from the horizontal and vertical scans (i.e., left–right, right–left, down–up, and up–down motion) by binning spikes into 100-ms bins, corresponding to 5° at each location on the two-dimensional display to produce a three-dimensional spike histogram represented on a false color plot. Receptive fields of non–direction-selective neurons were then averaged across all four scan directions. When neurons showed evidence of direction selectivity, we further analyzed the local preferred direction using a method analogous to that of Krapp and Hengstenberg [22]. The response to the four directions of motion at each point in the receptive field was fitted in a least squares manner with a sinusoid with variable phase, amplitude, and offset but a fixed frequency of 360°. We then used the phase of the fitted function to interpolate the local preferred direction and its relative amplitude (i.e., after normalizing for the overall maximum response of the neuron) to determine the length of local vectors.

Once receptive fields were determined, spike rates in subsequent experiments were averaged from the period during which targets traversed the receptive field. As spike rates varied between neurons, the responses were normalized to results of a standardized experiment in which the 0.8° target was moved against a bright background, in the preferred direction, and within the receptive field (Figure 7). When several repeats were performed in a neuron, the mean of the responses was calculated to give one value, which was averaged across several neurons in the pooled data sets (*n* = 8 forFigure 7A,*n* = 7 for 7B,*n* = 8 for 7C,*n* = 4 for 7D,*n* = 5 for 7E, and*n* = 3 for 7F). Significance was calculated with Student's paired 2-tailed*t-*test.

### Modeling

We developed an optical model in Matlab that permitted us to estimate the apparent contrast of targets smaller than the receptive field of individual photoreceptors. The model integrated the response across an assumed circular symmetrical receptive field of the photoreceptor, based on optical parameters derived from earlier work on fly photoreceptors and taking into account the waveguide properties [23–25]. Based on direct measures (kindly provided by Eric Warrant, University of Lund) of inter-ommatidial angle and ommatidial lens diameter in*Eristalis* males, our model utilized a Gaussian blur with a half-width of 1.1°. Since the targets modeled were black, with a theoretical contrast of 1.0, we expressed the integrated output of the receptive field relative to this value, for targets of different length and a constant width of 0.8° (as used in physiological experiments). The model thus gives a measure of apparent maximum (“neural”) contrast between a photoreceptor with a receptive field centered on the target and those viewing the background.

## Abbreviations

HS: horizontal system
STMD: small-target motion detector

## References

[pbio-0040054-b001] Gaffney MF, Hodos W (2003). The visual acuity and refractive state of the American kestrel*(Falco sparverius)*. Vision Res.

[pbio-0040054-b002] Giurfa M, Menzel R (1997). Insect visual perception: complex abilities of simple nervous systems. Curr Opin Neurobiol.

[pbio-0040054-b003] Collett T, King AJ, Horridge G (1975). Vision during flight. The compound eye and vision of insects.

[pbio-0040054-b004] Collett TS, Land MF (1975). Visual control of flight behaviour in the hoverfly,Syritta pipiens L. J Comp Physiol.

[pbio-0040054-b005] Collett TS, Land MF (1978). How hoverflies compute interception courses. J Comp Physiol A.

[pbio-0040054-b006] Stubbs AE, Falk SJ (1996). British hoverflies.

[pbio-0040054-b007] Collett T (1971). Visual neurones for tracking moving targets. Nature.

[pbio-0040054-b008] O'Carroll D (1993). Feature-detecting neurons in dragonflies. Nature.

[pbio-0040054-b009] Gilbert C, Strausfeld NJ (1991). The functional organization of male-specific visual neurons in flies. J Comp Physiol.

[pbio-0040054-b010] Egelhaaf M (1985). On the neuronal basis of figure-ground discrimination by relative motion in the visual system of the fly. II. Figure-detection cells, a new class of visual interneurones. Biol Cybern.

[pbio-0040054-b011] Warzecha AK, Egelhaaf M, Borst A (1993). Neural circuit tuning fly visual interneurons to motion of small objects. I. Dissection of the circuit by pharmacological and photoinactivation techniques. J Neurophysiol.

[pbio-0040054-b012] Hausen K, Egelhaaf M, Stavenga DG, Hardie RC (1989). Neural mechanisms of visual course control in insects. Facets of vision.

[pbio-0040054-b013] Farrow K, Haag J, Borst A (2003). Input organization of multifunctional motion-sensitive neurons in the blowfly. J Neurosci.

[pbio-0040054-b014] Harris RA, O'Carroll DC, Laughlin SB (2000). Contrast gain reduction in fly motion adaptation. Neuron.

[pbio-0040054-b015] Borst A, Haag J (2002). Neural networks in the cockpit of the fly. J Comp Physiol A.

[pbio-0040054-b016] Kandil FI, Fahle M (2004). Figure-ground segregation can rely on differences in motion direction. Vision Res.

[pbio-0040054-b017] Egelhaaf M, Hausen K, Reichardt W, Wehrhahn C (1988). Visual course control in flies relies on neuronal computation of object and background motion. Trends Neurosci.

[pbio-0040054-b018] Land MF, Eckert H (1985). Maps of the acute zones of fly eyes. J Comp Physiol A.

[pbio-0040054-b019] O'Carroll DC, Bidwell NJ, Laughlin SB, Warrant EJ (1996). Insect motion detectors matched to visual ecology. Nature.

[pbio-0040054-b020] Kelly DH (1979). Motion and vision. II. Stabilized spatio-temporal threshold surface. J Opt Soc Am.

[pbio-0040054-b021] Yeates DK, Dodson GN (1990). The mating system of a bee fly*(Diptera: Bombyliidae)*. I. Non-resource-based hilltop territoriality and a resource-based alternative. J Insect Behav.

[pbio-0040054-b022] Krapp HG, Hengstenberg R (1997). A fast stimulus procedure to determine local receptive field properties of motion-sensitive visual interneurons. Vision Res.

[pbio-0040054-b023] Smakman J, van Hateren J, Stavenga DG (1984). Angular sensitivity of blowfly photoreceptors: Intracellular measurements and wave-optical predictions. J Comp Physiol A.

[pbio-0040054-b024] Stavenga DG (2003). Angular and spectral sensitivity of fly photoreceptors. I. Integrated facet lens and rhabdomere optics. J Comp Physiol A.

[pbio-0040054-b025] van Hateren JH, Stavenga DG, Hardie RC (1989). Photoreceptors optics, theory and practics. Facets of vision.

